# Diagnostic Value of the Anal Endoluminal Functional Luminal Imaging Probe for the Diagnosis of Anismus: An Exploratory Study

**DOI:** 10.1111/nmo.70452

**Published:** 2026-10-02

**Authors:** Charlotte Desprez, Thomas Duflot, Chloé Melchior, Guillaume Gourcerol, Ali Zifan, Estelle Houivet, Céline Savoye‐Collet, Anne‐Marie Leroi

**Affiliations:** ^1^ Department of Digestive Physiology Université of Rouen Normandy, INSERM, ADEN UMR1073, CHU Rouen, CIC‐CRB 1404 Rouen France; ^2^ Department of Pharmacology University of Rouen Normandy, UNIROUEN, Inserm U1096, CHU Rouen, CIC‐CRB 1404 Rouen France; ^3^ Department of Gastroenterology University of Rouen Normandy, INSERM, ADEN UMR1073, CHU Rouen, CIC‐CRB 1404 Rouen France; ^4^ Division of Gastroenterology, Department of Medicine University of California La Jolla California USA; ^5^ Department of Biostatistics and Clinical Research CHU Rouen Rouen France; ^6^ Department of Radiology University of Rouen Normandy, AIMS, CHU Rouen Rouen France

**Keywords:** anal function, constipation, CSA‐pressure loop, FLIP, push maneuver

## Abstract

**Background and Aims:**

The aim of this exploratory study was to evaluate the performance of the anal EndoFLIP in diagnosing anismus in patients with functional constipation and suspected to have a dyssynergic defecation based on a composite reference standard (anorectal manometry, surface electromyography, and defecography).

**Methods:**

Patients underwent anorectal manometry, surface electromyography, and defecography. At least two of the three tests needed to be positive to diagnose anismus. Anal EndoFLIP was performed during push maneuvers at 40 and 50 mL of distension, and pressure‐cross‐sectional‐area (CSA) loops were built for each push maneuver to identify parameters that could best predict a diagnosis of anismus (ROC curve analysis). Two‐variable CSA–pressure classifiers were evaluated using nested repeated five‐fold cross‐validation.

**Results:**

Diagnosis of anismus using the composite reference standard was made in 29/57 patients (50.8%). An analysis of CSA‐pressure loops indicated that, at 40 mL of distension, maximal absolute ΔCSA and maximal relative ΔCSA were lower compared to patients without anismus (*p* < 0.001). Among quantitative CSA indices, an exploratory threshold under 12.8 mm^2^ for a maximal absolute ΔCSA at 40 mL provided the highest accuracy and AUC (0.81) and excellent specificity (0.93) for the diagnosis of anismus. Using nested repeated cross‐validation, two‐variable GLM and CART classifiers yielded mean out‐of‐sample AUCs of 0.767 and 0.764 at 40 mL and 0.754 and 0.658 at 50 mL, respectively.

**Conclusion:**

The results suggest that anal EndoFLIP may help distinguish patients with anismus from those without. Further external validation studies are needed to establish clinically applicable thresholds.

**Trial Registration:**

Clinical.trials.gov: NCT04155307

## Key Points

**Background:**

The aim of this exploratory study was to evaluate the performance of anal EndoFLIP in diagnosing anismus, compared with an unvalidated composite reference standard comprising anorectal manometry, defecography, and anal electromyography.

**Findings:**

An exploratory threshold under 12.8 mm^2^ for maximal absolute ΔCSA of the anal canal at 40 mL provided the highest accuracy and AUC (0.81) as well as excellent specificity (0.93) for the diagnosis of anismus.

**Implications for Patient Care:**

The results suggest that anal EndoFLIP may help distinguish patients with anismus from those without. Further confirmatory studies are needed to establish clinically applicable thresholds.

## Plain Language Summary

This study evaluated whether the anal EndoFLIP device can help diagnose anismus, a condition in which the muscles involved in bowel movements do not coordinate properly. Current guidelines recommend using at least two different tests to confirm the diagnosis.

This exploratory study found that EndoFLIP was effective at identifying patients with anismus and identified exploratory thresholds. Specifically, a maximum maximal absolute ΔCSA below 12.8 mm^2^ at a 40 mL measurement provided the best diagnostic performance. The test showed good overall accuracy and was particularly strong at correctly ruling out people who did not have the condition.

These results suggest that anal EndoFLIP could be a useful tool for distinguishing patients with anismus from those without it. Further confirmatory studies are needed to establish clinically applicable thresholds.

## Graphical abstract

The anal EndoFLIP exhibited a good ability to distinguish patients with anismus from those without based on the composite reference standard (anorectal manometry, surface electromyography, and defecography).

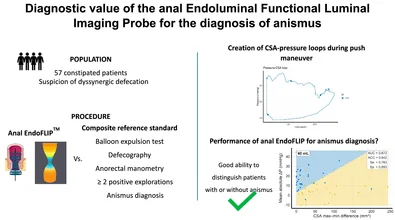

## 1 Introduction

Chronic constipation is the most common gastrointestinal disorder and leads to significant health care and social burdens [1]. The Rome V criteria categorize bowel and anorectal disorders into several diagnoses, including dyssynergic defecation (previously named “functional defecation disorders”) [2, 3]. This latter diagnosis requires the presence of symptoms suggestive of difficult evacuation and may satisfy diagnosis criteria for chronic constipation or irritable bowel syndrome, in association with features of impaired rectal evacuation as demonstrated by any one of the three following tests: anorectal manometry, balloon expulsion test, and defecography [4]. Patients with dyssynergic defecation can exhibit ineffective propulsion and/or features of anismus (i.e., paradoxical contraction or inadequate relaxation of the anal canal) [5]. When first‐line medical management fails, it is important to identify the subtype of constipation present to guide further treatment of the patient. For example, diagnosing anismus can lead to the implementation of specific treatments such as biofeedback, even though their efficacy remains controversial [6, 7].

Although symptoms and clinical examination may suggest anismus patterns, they do not reliably consistently distinguish patients with anismus from those without [4, 8]. Physiologic studies, including anorectal manometry (ARM), balloon expulsion test, surface anal electromyography (EMG), and defecography should be considered to help diagnose anismus. However, agreement between these tests remains limited [4]. Consequently, multiple complementary investigations are required to determine whether anismus is present [5]. Furthermore, the balloon expulsion test (BET) primarily assesses the overall ability to evacuate a rectal balloon but does not provide detailed physiological information on anorectal dynamics during simulated defecation, particularly regarding anal canal opening. A recent study observed that a reduced rectoanal gradient on ARM was often associated with a dyssynergic pattern of defecation and was the best predictor of prolonged BET or reduced rectal evacuation on defecography [9]. Accordingly, the Rome V criteria recommend measuring the rectoanal gradient to assess the quality of rectal evacuation [2]. However, a reduced rectoanal gradient may reflect inadequate propulsive forces and/or impaired anal relaxation. To date, the rectoanal gradient has not been demonstrated to be a reliable diagnostic criterion for anismus [2].

The Endoluminal Functional Lumen Imaging Probe (EndoFLIP, Crospon, Galway, Ireland) is a novel instrument that can measure the cross‐sectional area (CSA) along a sphincter during volumetric distension and assess sphincter distensibility [10, 11]. The EndoFLIP has been evaluated to assess the distensibility of the anal canal in healthy volunteers (HVs) [12, 13] and in patients with fecal incontinence [14, 15, 16], and appears to be superior to ARM for distinguishing between those two groups of subjects [17]. We recently evaluated anal canal opening by building CSA‐pressure loops during push maneuvers in HVs and uncovered potential markers of interest [16]. The aim of this exploratory study was to evaluate, in a population of constipated patients, the ability of EndoFLIP to distinguish between patients with anismus and those without compared to an anismus diagnosis based on the results of 3‐dimensional high‐resolution ARM (3D‐HRM), anal EMG, and defecography (unvalidated composite reference standard). As there are currently no normative values available for EndoFLIP in this context, we first visually assessed the opening of the anal canal during push maneuver on EndoFLIP recordings to diagnose anismus. Next, we employed a novel approach involving CSA‐pressure loops during push maneuver to identify various anal canal opening parameters that could be used to diagnose anismus in a population of patients with constipation.

## 2 Methods

### 2.1 Patients

Patients with a diagnosis of functional constipation according to Rome IV criteria [18] and a suspected dyssynergic defecation were recruited at the Physiology Unit of Rouen University Hospital between January 2020 and March 2024 for the present prospective study. All the patients included in the study were over 18 years of age and exhibited delayed colonic transit study with most radio‐opaque markers found in the rectosigmoid area (21 h in men and 38 h in women), as previously published [19, 20]. Exclusion criteria included suspicion of slow‐transit constipation alone, inflammatory or infectious disease or carcinoma of the anorectal or colonic area, proctologic disease requiring dedicated treatment, anorectal malformation, history of pelvic radiation, history of colostomy, and contra‐indication to the placement of an endoanal probe. Quality of life was measured using Gastrointestinal Quality of Life Index (GIQLI) score ranging from 0 (worst quality of life) to 144 (best quality of life) [21]. The Kess score was used to evaluate anorectal symptoms [22]. All patients gave their written informed consent. The present study complied with the ethical principles of the Declaration of Helsinki, was approved by the Ile‐de‐France regional ethics committee (Study N°2018/347/HP, CPPIDF1‐2019‐ND41), and was registered with Clinical.trials.gov (NCT04155307).

### 2.2 Anorectal Functional Tests

Every patient underwent three pelvic floor examinations, including 3D‐HRM, defecography, and anal EMG. These anorectal physiological tests were used as a “composite reference standard” in the present study for the diagnosis of anismus, with at least two positive examinations among these three anorectal physiological tests required to confirm a definitive diagnosis of anismus. As we did not use BET in the present study to assess rectal evacuation, the composite reference standard differed from that used in Rome IV criteria. These tests were performed on the same day in the same order for all patients (anal EndoFLIP first, followed by 3D‐HRM and anal EMG, with 1.5‐h intervals between each test), except for defecography, which was performed on a separate day after a maximum delay of 3 weeks.

#### 2.2.1 Anorectal Manometry

3D‐HRM was performed as previously described using a solid‐state probe (AR 3D probe Manoscan, Medtronic Inc., Minneapolis, MN, USA) [23]. Manometric and topographic images are displayed using a dedicated software (ManoView, Medtronic Inc., Minneapolis, MN, USA). Three push maneuvers were performed during recordings separated by 30 s with a duration of 15 s each, as described by the IAPWG protocol [5]. The best attempt among the three maneuvers was used for analysis. Normal ranges for each parameter were obtained from 40 female healthy volunteers and are described elsewhere [23]. Briefly, normal ranges used were: maximal increase in intrarectal pressure during push maneuver > 13 mmHg, anal resting pressure (58–90 mmHg), and a reduction of more than 20% in anal pressure during the push maneuver [5]. Anorectal pressures at baseline and during push maneuvers were used for the diagnosis of anismus using the London Classification as follows [5]: faulty anal relaxation during the push maneuver (less than 20% of reduction in anal pressure or a paradoxical anal contraction). Subtypes of dyssynergic defecation as described by Rao et al. were also used to describe the results of 3D‐HRM [4].

#### 2.2.2 Barium Defecography

Patients underwent defecography using established approaches [9, 24]. The criteria for an abnormal finding suggesting a diagnosis of anismus were the result of impaired rectal evacuation (expelled barium product < 25%) [9] in association with one of the following criteria: poor pelvic floor relaxation (maximal lower anal canal width < 0.5 cm), persistent imprint of the puborectalis muscle, and/or closing of the anorectal angle during push maneuver [24].

#### 2.2.3 Anal Electromyography

Anal electromyography was performed using single‐use concentric needle electrodes (Ambu Neuroline Concentric, Ambu A/S, Ballerup, Denmark) connected to an electromyography system equipped with a Nicolet AT2+6 amplifier (Nicolet EDX/Viking EDX system, Natus Neurology Incorporated, Middleton, WI, USA) as previously described [25]. Anal EMG activity was subsequently recorded during repeated simulated defecation attempts. Anismus was suspected following a change of less than 20% of baseline values or a paradoxical increase in electrical activity [26].

### 2.3 EndoFLIP Assessment

In addition to the previously described anorectal physiological tests, all patients underwent anal EndoFLIP (8‐cm balloon, Catheter EF‐325N; Crospon Ltd., Galway, Ireland) for the assessment of anal canal opening during a push maneuver as previously described [16]. Air was removed from the probe before data acquisition and the baseline intra‐bag pressure was zeroed to atmospheric pressure. Patients were placed in a lateral decubitus position and the probe was introduced, under visual inspection, until only two detection electrodes remained visible outside the anal verge. The bag was filled with conductive saline solution at a rate of 40 mL/min from 0 to 50 mL of distension at 10‐mL steps. At 40 and 50 mL of distension, the patients were asked to perform three push maneuvers with resting periods of 30 s between each. The best attempt (e.g., the most qualitatively normal with best opening of anal canal) was kept for analysis. CSA and pressure measurements were acquired at 10 Hz. The probe was held in place manually during the acquisitions and repositioning could occur between push maneuvers.

### 2.4 Data Analysis

Software specifically designed to display EndoFLIP data (EndoVizX, Motilityviz LLC, La Jolla, CA, USA) was used to display recordings of CSAs and intra‐bag pressure (P) at 40 mL and 50 mL of distension at rest and during push maneuver. Temporal variations in diameter along the EndoFLIP probe were represented as a heatmap (small diameters in red, large diameters in blue) as were temporal variations in intra‐bag pressure and distension volumes. The operator's diagnosis regarding the absence or presence of anismus (e.g., visual diagnosis of anismus) was based solely on a visual inspection of the anal canal opening on the EndoFLIP recording. Performance of the EndoFLIP and diagnosis of anismus were blinded from clinical history and results of the other anorectal examinations. Two experienced operators (CD and AML), who were blinded to each other and from results of other anorectal explorations, assessed the EndoFLIP recordings for a visual diagnosis of anismus. In the event of disagreement, the EndoFLIP recording was visualized in the presence of the two operators to reach a consensus regarding the diagnosis of anismus. Inter‐rater agreement was assessed using calculation of Cohen's kappa coefficient. The EndoFLIP results were then compared with the diagnosis of anismus based on the defecography, anorectal manometry and electromyogram results where two of the three tests had to be positive to make the final diagnosis. When a patient did not undergo all three tests, the following criteria were applied to determine the presence of constipation with anismus:

– When two of the three tests were conducted and both were negative for the presence of constipation with anismus, the patient was classified as not having constipation with anismus
– When two of the three tests yielded positive results for constipation with anismus, the patient was diagnosed with constipation with anismus.
– When a single test was conducted, or if two of the three tests were conducted and one was positive and the other one was negative for constipation with anismus, it was not possible to draw a conclusion. In such cases, the variable was classified as missing data.

For each distension volume, we computed a confusion matrix against the reference using the caret [27] package for R version 4.4.2 [28]. The following metrics were extracted: accuracy, sensitivity (recall), specificity, positive predictive value (PPV), and negative predictive value (NPV).

We then analyzed the CSA and temporal pressure changes at 40 mL and 50 mL of distension as predictors of anismus. For every patient, CSA‐pressure loops during a push maneuver were built at 40 mL and 50 mL of distension using EndoVizX software (Figure 1), as previously described [16]. Briefly, the software was used to isolate and extract data of the best push maneuver at each distension volume using the “region of interest” function of the software, and the minimal diameter and the intra‐bag pressure values were collected for each acquisition (10‐Hz frequency). These analyses and data extraction were performed while blinded to the results of the other anorectal investigations. The lowest CSA along the probe was then computed for each acquisition from the lowest diameter with a circular approximation [29]. Initial CSA and pressure values of the EndoFLIP recordings were used as baseline values to assess variations during the push maneuver (= baseline CSA and P). Specifically, for each volume (i.e., 40 mL and 50 mL of distension), we computed the maximal absolute difference from baseline corresponding to the CSA at maximal distension minus the baseline CSA (Max Absolute ΔCSA) and the maximal relative difference in percentage from baseline corresponding to the CSA at maximal distension divided by the baseline CSA (Max Relative ΔCSA), which resulted in four predictors. For each of the four CSA predictors, we performed a receiver operating characteristic (ROC) curve analysis against the binary outcome (anismus yes/no) using the pROC [30] package.

**FIGURE 1:**
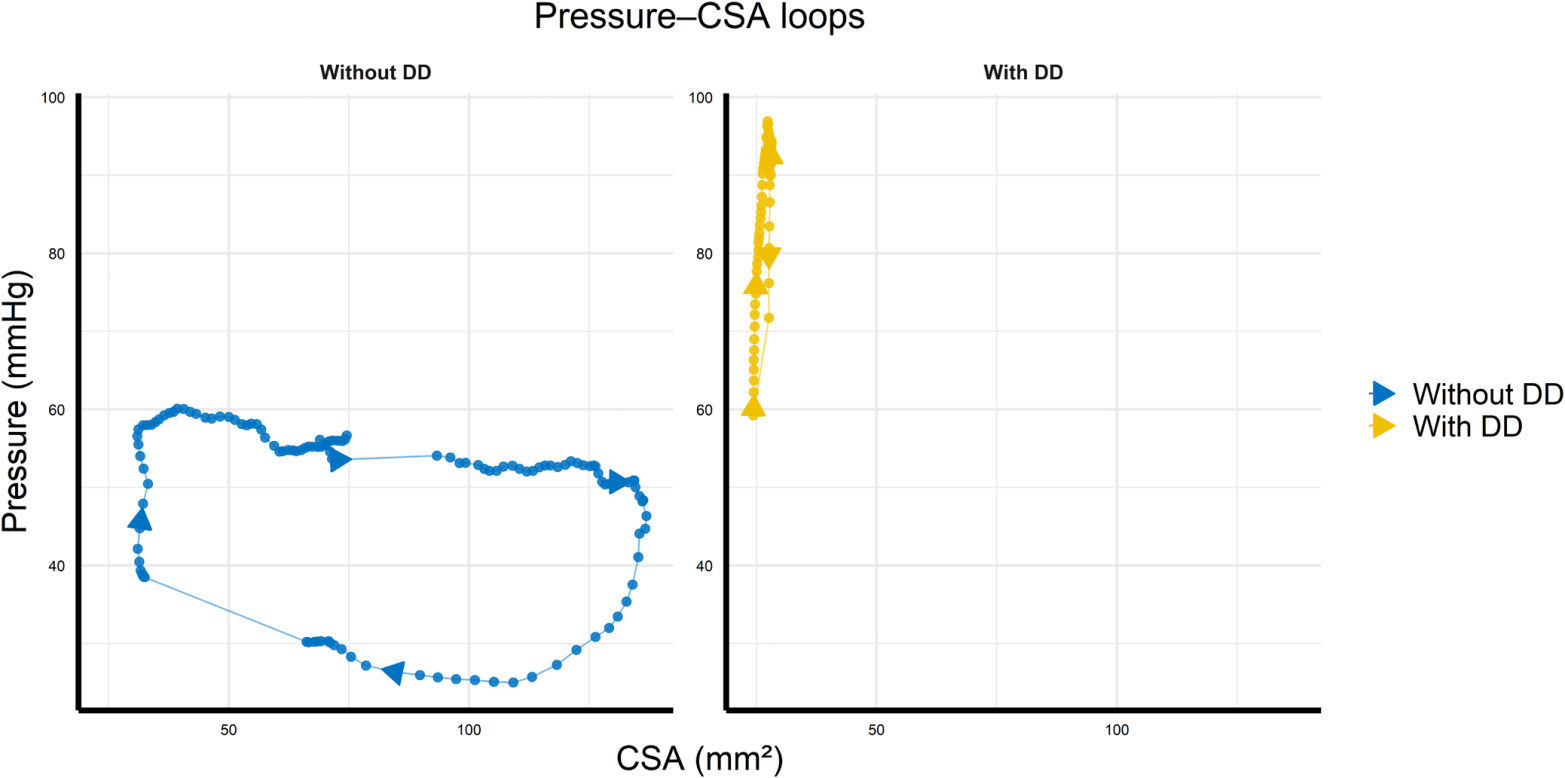
Examples of CSA‐pressure loops at 40 mL of distension to evaluate sphincter opening as a function of time during the push maneuver from a patient without anismus (blue loop) and from a patient with anismus and markedly impaired anorectal biomechanical properties (yellow loop). CSA, cross‐sectional‐area.

The analytical steps included the computation of the area under the ROC curve (AUC) with 2000 stratified bootstrap replicates to derive a percentile‐based 95% confidence interval and to identify the threshold maximizing Youden's index. We recorded its sensitivity, specificity, positive predictive value (PPV), negative predictive value (NPV), and accuracy. We visualized all four ROC curves on a single figure using ggplot2 [31] and applied a custom theme for clarity. The error bars around each optimal threshold point represent 95% CIs for sensitivity (vertical) and specificity (horizontal) and were “nudged” to lie on the smoothed curve.

In addition to the CSA‐based predictors, we also evaluated a comprehensive exploratory set of secondary parameters derived directly from the CSA‐pressure loops during push maneuvers at both 40 mL and 50 mL. In total, this yielded the following 12 parameters for pressure and 12 parameters for CSA at both distension volumes (48 parameters in total):

– Baseline P or CSA: Baseline value of pressure (mmHg) or CSA (mm^2^), corresponding to the plateau value of P or CSA immediately before the push maneuver
– Min P or CSA: Minimal value of pressure (mmHg) or CSA (mm^2^) during the push maneuver. Minimal CSA: if a patient exhibited paradoxical closure of the anal canal during the push maneuver, this parameter was inferior to baseline CSA and reflected a decrease in CSA. This could not be computed for every patient
– Max P or CSA: Maximal value of pressure (mmHg) or CSA (mm^2^) during the push maneuver
– Mean P or CSA: Mean value of pressure (mmHg) or CSA (mm^2^) during the push maneuver
– Max absolute ΔP or ΔCSA: Maximal absolute variation in pressure (ΔmmHg from baseline) or in CSA (Δmm^2^ from baseline) computed from baseline
– Max relative ΔP or ΔCSA: Maximal relative variation in pressure (% of baseline) or in CSA (% of baseline) computed from baseline
– Mean absolute ΔP or ΔCSA: Mean absolute variation in pressure (ΔmmHg from baseline) or in CSA (Δmm^2^ from baseline) computed from baseline
– Mean relative ΔP or ΔCSA: Mean relative variation in pressure (% of baseline) or in CSA (% of baseline) computed from baseline
– Absolute P or CSA min‐baseline difference (ΔmmHg or Δmm^2^ from baseline): difference between baseline and minimal value of P or CSA during push maneuver. If a patient exhibited paradoxical closure of the anal canal during the push maneuver, minimal CSA was inferior to baseline CSA and reflected a decrease in CSA. This could not be computed for every patient.
– Relative P or CSA min‐baseline difference (% from baseline)
– Absolute P or CSA max‐min difference (ΔmmHg or Δmm^2^ from minimal): difference between maximal and minimal value of P or CSA during push maneuver
– Relative P or CSA max–min difference (% from minimal)

Continuous CSA‐ and pressure‐derived parameters are presented as mean ± standard deviation overall and according to anismus status. Between group comparisons were performed using the Welch's two‐sample *t*‐test. Because multiple correlated parameters were explored at two distension volumes, raw *p* values were adjusted across all comparisons reported in Table 3 using the Benjamini‐Hochberg procedure to control the false discovery rate. Statistical significance was defined as an FDR‐adjusted *p* value (*q*‐value) < 0.05. Given the exploratory nature of the analysis and the correlation among several derived parameters, the FDR‐adjusted results should be interpreted as hypothesis‐generating and require confirmation in an independent cohort. We also computed ROC curve characteristics (AUC, threshold, sensitivity, specificity, accuracy).

Lastly, we investigated whether combining one CSA‐derived and one pressure‐derived parameter could improve discrimination of anismus. At each distension volume, 12 CSA‐derived and 12 pressure‐derived variables yielded 144 candidate pairs. Two‐variable GLM and CART models were evaluated using nested repeated cross‐validation, with an outer stratified five‐fold cross‐validation repeated 100 times. Within each outer training set, all 144 pairs were compared using an inner stratified five‐fold cross‐validation, and the pair with the highest inner out‐of‐fold AUC was selected (Brier score as tie‐breaker). The selected model was then refitted on the complete outer training set and evaluated on the held‐out outer fold. CART models were constrained to two sequential splits and to use both the CSA‐ and pressure‐derived predictors. Diagnostic performance was estimated exclusively from outer out‐of‐fold predictions and the most frequently selected pair for each model and volume was refitted to the complete dataset.

According to the literature, the percentage of patients with both constipation and functional defecation disorders is 47% [31]. Given that ours was a pilot study with a sample size of 60 patients, we expected to have 30 patients with anismus and 30 patients without anismus. This sample size would have allowed us to estimate the sensitivity of the test with such precision that the width of the confidence interval for sensitivity would be no more than ±17.9% around the estimated percentage (width obtained if the sensitivity was 50%, which represented the worst possible scenario). For greater sensitivity, for example, approximately 90%, the precision would be ±10.7% with the same sample size.

All the authors had access to the study data and reviewed and approved the final manuscript.

## 3 Results

### 3.1 Patients

Sixty‐one patients (56/61 [91.8%] female gender, median age 49.1 [33.9; 58.7] years) were considered for study inclusion. All the patients underwent anal EMG, 60/61 underwent 3D‐HRM, and 54/61 underwent defecography. Patients' characteristics and the results of anorectal physiological tests are summarized in Table 1. Flow chart of the study is given in Figure 2. Anismus was diagnosed by 3D‐HRM in 39/60 (65.0%) of patients, by anal EMG in 30/61 (49.2%) patients and by defecography in 12/54 (22.2%) patients. Fifty‐seven patients were included in statistical analysis (Figure 2). A definitive diagnosis of constipation with anismus was confirmed in 29/57 (50.8%) patients (Table 1, Figure 2). Among patients having a definitive diagnosis of anismus according to the gold standard, 11 patients (37.9%) had a positive defecography, 26 patients (89.7%) had a positive 3D‐HRM, and 27 patients (93.1%) had a positive anal EMG. Of the three patients excluded from the analysis due to conflicting anismus diagnosis results between 3D‐HRM and anal EMG, two were suspected of having anismus based on anal EMG results and one based on 3D‐HRM results.

**TABLE 1:** Patients characteristics and results of anorectal examinations (high‐resolution anorectal manometry, anal electromyography and defecography).

Parameters	All patients (*N* = 61)
Age (years)	49.1 [33.9; 58.7]
Female gender, *n* (%)	56 (91.8)
BMI	21.8 [19.9; 25.7]
KESS score	20.5 [9.0; 25.8]
GIQLI score	90.0 [78.3; 100.8]
Anorectal manometry	(*N* = 60)
Maximal increase in rectal pressure during push maneuver (mmHg)	32 [20; 45]
Decreased rectal pressure during push maneuver, *n* (%)	2 (3.3)
Anal resting pressure (mmHg)	72 [57; 89.5]
Increased anal pressure during push maneuver, *n* (%)	27 (45.0)
Reduced anal relaxation, *n* (%)	12 (20.0)
Type of dyssynergic defecation evidenced on anorectal manometry	
Type I, *n* (%)	26 (45.6)
Type II, *n* (%)	1 (1.8)
Type III, *n* (%)	10 (17.5)
Type IV, *n* (%)	1 (1.8)
Anismus, *n* (%)	39 (65.0)
Anal electromyography	(*N* = 61)
Anismus, *n* (%)	30 (49.2)
Defecography	(*N* = 54)
Rectocele	9 (15.7)
Intussuception	2 (3.5)
Enterocele	1 (1.8)
Reduced anal canal opening, *n* (%)	7 (13.0)
Reduced evacuation < 25%, *n* (%)	13 (24.1)
Closing of anorectal angle, *n* (%)	10 (18.5)
Persistent imprint of puborectalis muscle, *n* (%)	10 (18.5)
Anismus, *n* (%)	12 (22.2)
Constipation with anismus, *n* (%)	29 (50.0)

*Note:* Results are given as median (IQ 25th–75th).

Abbreviation: BMI, body mass index.

**FIGURE 2:**
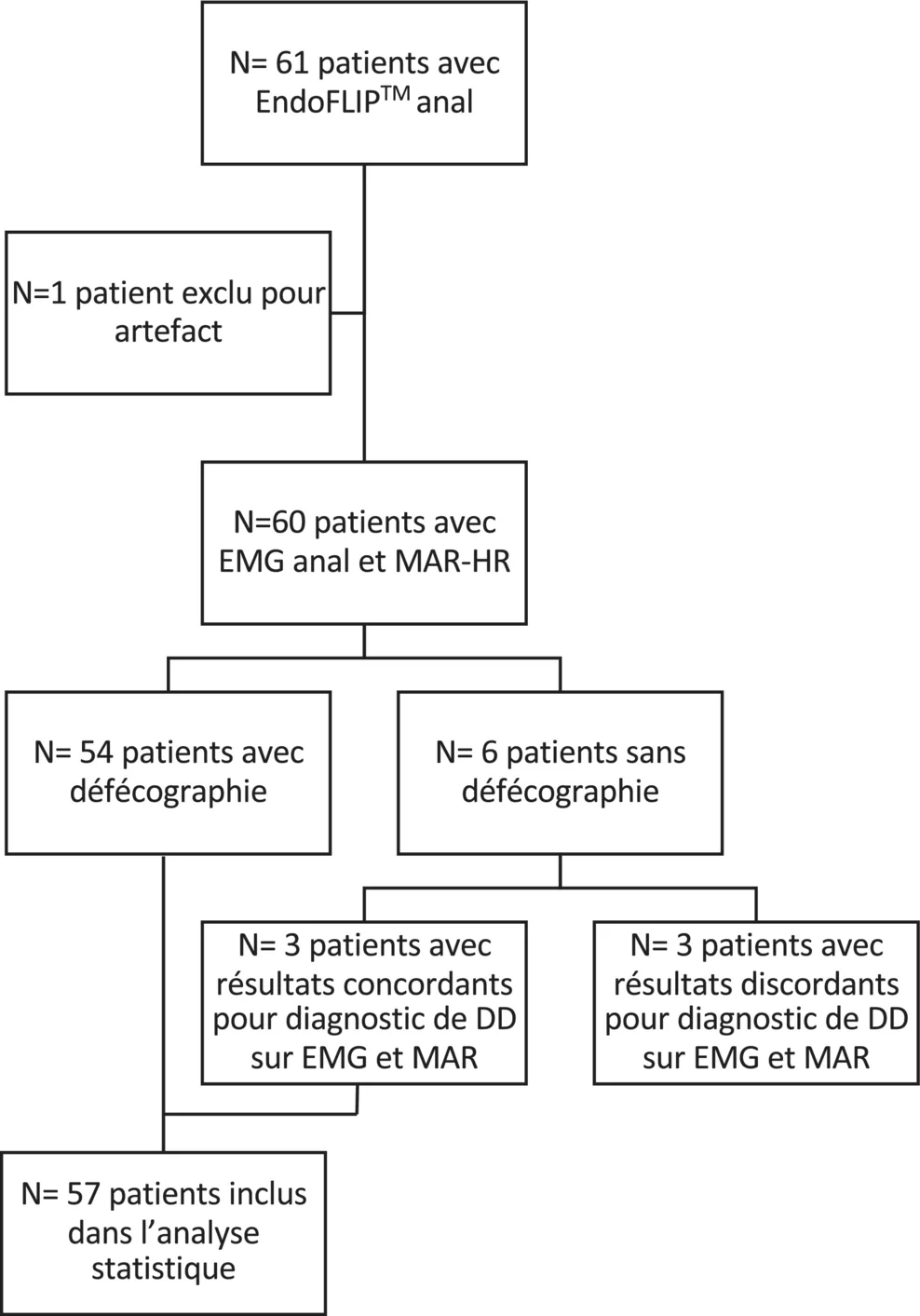
Flow‐chart of the study. EMG, electromyography; HR‐ARM, high‐resolution anorectal manometry.

### 3.2 Anal EndoFLIP Results: Visual Diagnosis of Anismus Compared With the Composite Reference Standard

Sixty‐one patients underwent anal EndoFLIP: one recording was excluded from the analysis of the push maneuvers due to an artifact and three recordings were excluded due to an inconclusive diagnosis of anismus by the composite reference standard (Figure 2). Fifty‐seven patients were included in statistical analysis. At 40 mL of inflation, the median duration of the push maneuver was 12.4 s (IQR 25–75: 10.0–15.2), and at 50 mL of inflation, it was 12.0 s (IQR 9.3–16.0). A visual diagnosis of anismus was made in 30 (52.6%) patients at 40 mL of distension and in 26 (45.6%) patients at 50 mL of distension (Figure 3). Cohen's kappa coefficients were 0.86 (95% CI: 0.73–0.99) at 40 mL of distension and 0.86 (95% CI: 0.78–1.00) at 50 mL of distension before consensus. Anal EndoFLIP contingency tables for the diagnosis of anismus at 40 mL and 50 mL of distension compared to the composite reference standard are given in Table 2. Visual assessments yielded the same overall accuracy at both distension volumes (73.7%) with a balanced sensitivity (40 mL = 75.9%, 95% CI [57.9; 87.8]; 50 mL = 69.0%, 95% CI [50.8; 82.7]) and specificity (40 mL = 71.4%, 95% CI [53.0; 84.8]; 50 mL = 78.6%, 95% CI [60.5; 89.8], Table 2).

**FIGURE 3:**
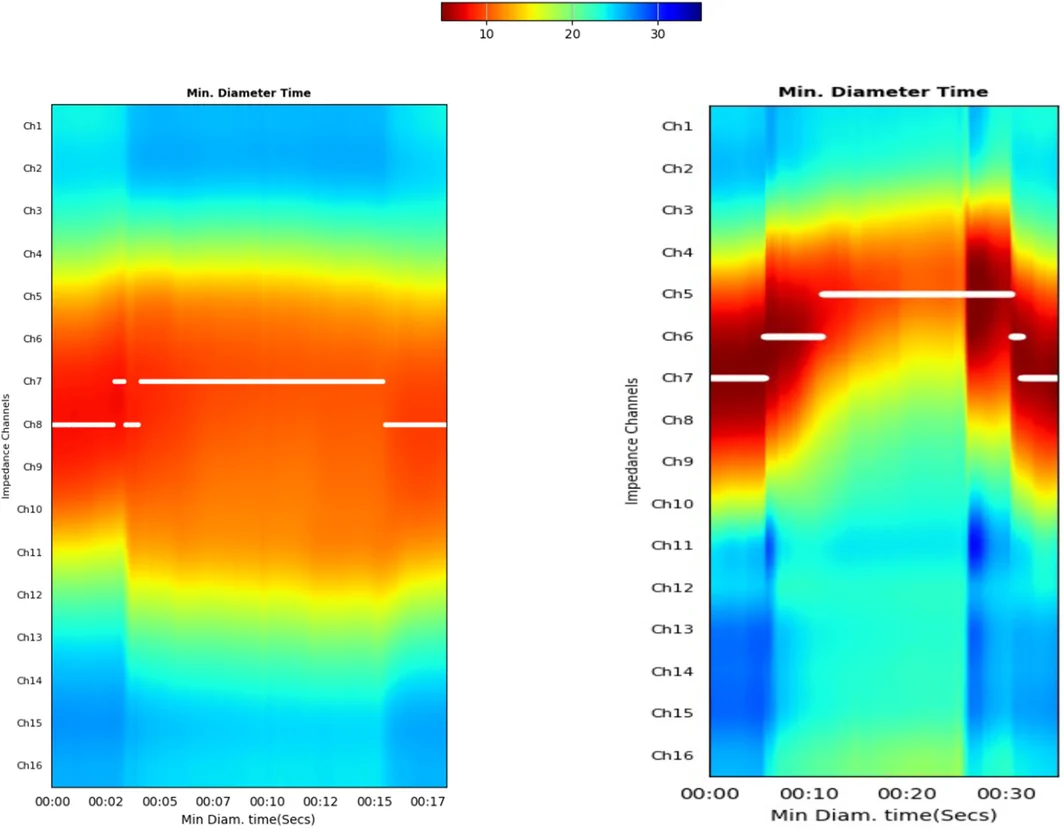
EndoFLIP recordings of push maneuvers using the ROI function at 40 mL of distension in a patient with anismus (left) and in a patient without anismus (right). Displays of the “minimal diameter” function (horizontal white line) to visualize the location of the lowest diameter during each acquisition during the push maneuver in two patients. Temporal diameter changes along the EndoFLIP probe are shown as a heatmap (small diameters in red, large diameters in blue).

**TABLE 2:** Anal EndoFLIP contingency table for the diagnosis of anismus compared with the composite reference standard (at least 2 tests with a diagnosis of anismus among HR‐ARM, anal EMG, and defecography) at 40 mL (A) and 50 mL (B) of distension.

(A)			
		Composite reference standard (HR‐ARM, anal EMG, defecography)	
		Definitive diagnosis of anismus	Exclusion of anismus
Anal EndoFLIP	Visual diagnosis of anismus	22	8
	Exclusion of anismus	7	20

(B)			
		Composite reference standard (HR‐ARM, anal EMG, defecography)	
		Definitive diagnosis of anismus	Exclusion of anismus
Anal EndoFLIP	Visual diagnosis of anismus	20	6
	Exclusion of anismus	9	22

*Note:* (A) Sensitivity = 75.9%, 95% CI [57.9; 87.8]; Specificity = 71.4%, 95% CI [53.0; 84.8]; Positive predictive value = 73.3%, 95% CI [54.1; 87.7]; Negative predictive value = 74.07%, 95% CI [53.7; 88.9]; (B) Sensitivity = 69.0%, 95% CI [50.8; 82.7]; Specificity = 78.6%, 95% CI [60.5; 89.8]; Positive predictive value = 76.9%, 95% CI [56.4; 91.0]; Negative predictive value = 71.0%, 95% CI [51.9; 85.8].

Abbreviations: ARM, anorectal manometry; EMG, electromyography.

### 3.3 Comparison of CSA‐Pressure Loop Characteristics at 40 mL and 50 mL of Distension in Patients With or Without Anismus (Diagnosed by a Composite Reference Standard)

To determine whether the parameters of anal canal opening functions could discriminate patients with anismus from patients without, CSA‐pressure loops during push maneuvers were built for each patient at 40 mL and 50 mL of distension. The characteristics of patients' CSA‐pressure loop at 40 mL and 50 mL of distension are given in Table 3. After adjustment for multiple comparisons using the Benjamini‐Hochberg false discovery rate procedure, baseline CSA values (including baseline, minimal, maximal, and mean CSA values) were similar for patients with or without anismus at both 40 mL and 50 mL of distension (Table 3, Figure 1). Minimal pressures were higher at both 40 mL and 50 mL of distension in patients with anismus compared to patients without (45.5 ± 13.2 mmHg vs. 33.2 ± 9.1 mmHg and 55.7 ± 13.5 mmHg vs. 42.0 ± 9.9 mmHg respectively, *q* = 0.003; Table 3, Figure 1). Similar results were found for maximal pressure at both 40 mL and 50 mL of distension (75.2 ± 22 mmHg vs. 60.1 ± 11.8 mmHg, *q* = 0.023 and 85.3 ± 22 mmHg vs. 67.6 ± 14.5 mmHg respectively, *q* = 0.012, Table 3).

**TABLE 3:** Comparison of CSA‐pressure loop characteristics at 40 mL and 50 mL of distension in patients with or without anismus diagnosed by the composite reference standard.

Variable	Overall (40 mL)	Without anismus (40 mL)	With anismus (40 mL)	Overall (50 mL)	Without anismus (50 mL)	With anismus (50 mL)
Baseline CSA (mm^2^)	45 ± 37.6	47 ± 28.9	43 ± 44.8	133.6 ± 90.3	139.9 ± 74.8	127.5 ± 104.2
Minimal CSA (mm^2^)	42.5 ± 36	44.7 ± 26.6	40.5 ± 43.6	121 ± 83	134.1 ± 72	108.3 ± 91.8
Maximal CSA (mm^2^)	88.7 ± 78.8	111.1 ± 72.8	67.1 ± 79.6	199.8 ± 119.1	230.2 ± 102.9	170.4 ± 127.8
Mean CSA (mm^2^)	69.3 ± 59.2	83.8 ± 52.6	55.3 ± 62.8	165.6 ± 103.5	190 ± 90.4	142.1 ± 111.3
Max absolute ΔCSA (mm^2^)	43.7 ± 55.8	**64.1 ± 60.5**	**24.1 ± 43.4** *	66.1 ± 66.3	**90.3 ± 62**	**42.8 ± 62.8** *
Max relative ΔCSA (% of baseline)	109.8 ± 134.4	**156.5 ± 129.4**	**64.6 ± 125.3** *	79.2 ± 131.4	89.8 ± 94.1	69 ± 160.6
Mean absolute ΔCSA (mm^2^)	24.3 ± 33.3	**36.8 ± 36.6**	**12.3 ± 25** *	32 ± 43.9	**50 ± 40.8**	**14.6 ± 40.3** **
Mean relative ΔCSA (% of baseline)	59 ± 74.3	**86.6 ± 73.8**	**32.4 ± 65.6** *	36.5 ± 63.2	44.9 ± 37.4	28.3 ± 80.6
Absolute CSA min‐baseline difference (mm^2^)	−2.5 ± 6	−2.3 ± 5.9	−2.6 ± 6.2	−12.6 ± 21.6	**−5.8 ± 14**	**−19.3 ± 25.5** *
Relative CSA min‐baseline difference (% from baseline)	−4.7 ± 8.2	−4.2 ± 6.9	−5.2 ± 9.4	−9.2 ± 14.9	**−3.5 ± 7.5**	**−14.7 ± 18.1** *
Absolute CSA max–min difference (mm^2^)	46.2 ± 55.1	**66.4 ± 59.8**	**26.7 ± 42.8** *	78.8 ± 65.2	96.1 ± 60.8	62.1 ± 66.1
Relative CSA max–min difference (% from min)	118.5 ± 134.2	**165.7 ± 128.2**	**72.9 ± 125.7** *	97.2 ± 129.3	96.1 ± 92.3	98.3 ± 158.8
Baseline Pressure (mmHg)	45.4 ± 10.3	43.0 ± 7.7	47.6 ± 12	53.9 ± 11.2	**50.1 ± 8.9**	**57.6 ± 12.1** *
Minimal Pressure (mmHg)	39.5 ± 12.9	**33.2 ± 9.1**	**45.5 ± 13.2** **	49.0 ± 13.7	**42.0 ± 9.9**	**55.7 ± 13.5** ***
Maximal Pressure (mmHg)	67.8 ± 19.2	**60.1 ± 11.8**	**75.2 ± 22** **	76.6 ± 20.6	**67.6 ± 14.5**	**85.3 ± 22** ***
Mean Pressure (mmHg)	57.6 ± 17.2	**49.5 ± 11.1**	**65.4 ± 18.4** **	66.7 ± 17.8	**57.8 ± 11.3**	**75.3 ± 18.8** **
Max absolute ΔP (mmHg)	22.4 ± 14.7	**17.1 ± 12.4**	**27.5 ± 15.1** *	22.7 ± 15.7	**17.5 ± 14.7**	**27.7 ± 15.2** *
Max relative ΔP (% from baseline)	50.9 ± 33.5	43 ± 35.9	58.6 ± 29.6	42.9 ± 30.1	37.5 ± 33.7	48.2 ± 25.7
Mean absolute ΔP (mmHg)	12.2 ± 12.4	**6.5 ± 11.8**	**17.8 ± 10.5** **	12.8 ± 12.5	**7.7 ± 11.9**	**17.7 ± 11.2** **
Mean relative ΔP (% from baseline)	27.8 ± 28.6	**17.6 ± 31.7**	**37.6 ± 21.5** *	24.3 ± 24.2	17.6 ± 26.8	30.7 ± 19.8
Absolute P min‐baseline difference (mmHg)	−5.9 ± 7.2	**−9.8 ± 8.1**	**−2.1 ± 3.1** ***	−5 ± 6.7	**−8.1 ± 7.3**	**−1.9 ± 4.1** **
Relative P min‐baseline difference (% from baseline)	−13.6 ± 16.1	**−22.3 ± 17.8**	**−5.2 ± 8.1** ***	−9.8 ± 13.4	**−16.1 ± 14.8**	**−3.7 ± 8.4** **
Absolute P max–min difference (mmHg)	28.3 ± 13.4	26.9 ± 11.8	29.6 ± 14.8	27.6 ± 15	25.6 ± 14.4	29.6 ± 15.5
Relative P max‐min difference (% from min)	79.7 ± 46.2	91.6 ± 55	68.2 ± 32.6	61.4 ± 41.3	67.9 ± 49.7	55.2 ± 30.8

*Note:* Measurements are presented as mean ± sd for the overall population and stratified by dyssynergic defecation diagnosis, at 40 mL and 50 mL of distension. Between‐group comparisons were performed using Welch's two‐sample *t*‐test. *p* Values were adjusted across all comparisons included in Table 3 using the Benjamini–Hochberg false discovery rate procedure. Asterisks indicate FDR‐adjusted statistical significance: *q < 0.05, **q < 0.01, ***q < 0.001. Bold values indicate *p* < 0.05.

Abbreviations: CSA, cross‐sectional area; mm, millimeters; mmHg, millimeters of mercury.

Maximal absolute and maximal relative ΔCSAs at 40 mL of distension were lower in patients with anismus compared to patients without anismus, reflecting reduced anal canal opening in patients with anismus [24.1 ± 43.4 mm^2^ vs. 64.1 ± 60.5 mm^2^ (*q* = 0.032) and 64.6% ± 125.3% vs. 156.5% ± 129.4% (*q* = 0.032), Table 3, Figure 1]. The same results were observed for mean absolute and mean relative ΔCSAs at 40 mL of distension (Table 3). At 50 mL of distension, a significant decrease in ΔCSA was only observed for mean and maximal absolute ΔCSAs in patients with anismus in comparison to those without anismus (14.6 ± 40.3 mm^2^ vs. 50.0 ± 40.8 mm^2^, *q* = 0.006, and 42.8 ± 62.8 mm^2^ vs. 90.3 ± 62.2 mm^2^, *q* = 0.023, Table 3). In contrast, relative ΔCSA values did not differ significantly between groups at this volume (Table 3). Interestingly, at 50 mL of distension, relative CSA min‐baseline differences were higher in patients with anismus (−14.7% ± 18.1% vs. −3.5% ± 7.5%, *q* = 0.027, Table 3), pointing to some degree of anal canal closure in patients with anismus during the push maneuver. In addition, at 50 mL, patients with anismus showed a wider distribution of absolute CSA difference (baseline‐min) ranging from 0 to 75 mm^2^ and relative (baseline‐min)/min CSA ratios from 0.6 to 1.0, consistent with a more heterogeneous response and, in some patients, paradoxical anal canal closure (Figure S1). In contrast, at 40 mL of distension, the distributional spread of CSA variations between minimal and baseline values was similar in patients with and without anismus.

Maximal and mean absolute Δ*p* values were significantly higher in patients with anismus at both 40 and 50 mL of distension (Table 3). Mean relative Δ*p* was higher in patients with anismus at 40 mL (37.6% ± 21.5% vs. 17.6% ± 31.7%, *q* < 0.05, Table 3), whereas the difference at 50 mL of distension was no longer significant after FDR adjustment. No significant correlations were found between GIQLI or Kess scores and EndoFLIP parameters (CSA and pressure loop measurements) at either 40 or 50 mL of distension (Table S2).

### 3.4 Predictors of the Diagnosis of Anismus Among CSA‐Pressure Loops Characteristics

We evaluated the discriminative performance of 48 CSA‐pressure loop–derived parameters for distinguishing patients with anismus from others, using a ROC analysis (Table S1). Quantitative CSA indices performed markedly better than visual diagnoses of anismus. At 40 mL, the maximal absolute ΔCSA during push maneuver achieved the highest accuracy (0.81) and an AUC of 0.81, with a threshold of 12.8 mm^2^ providing excellent specificity (0.93) while still retaining 69% sensitivity (Figure 4). The maximal relative ΔCSA at the same volume ranked second (accuracy = 0.78; AUC = 0.80) when a discrimination cut‐off of 60.8% was used. At 50 mL the pattern was similar but slightly attenuated. The maximal absolute ΔCSA (threshold = 28.3 mm^2^) reached 77% accuracy (AUC = 0.78; sensitivity = 0.69; specificity = 0.86), whereas the maximal relative ΔCSA (threshold = 30.2%) matched the accuracy of visual inspections (0.74) with an AUC of 0.76 (Figure 4). In terms of pressure metrics, mean absolute ΔP at 40 mL reached a comparable AUC of 0.81 (threshold 10.5 mmHg), with a sensitivity of 82.8%, specificity of 75.0%, and an accuracy of 78.9%. In addition, the mean pressure (mmHg) at 50 mL displayed the highest AUC overall (0.82) and matched the 78.9% degree of accuracy (threshold 63.9 mmHg, sensitivity 79.3%, and specificity 78.6%, Table S1). Additional analyses were performed to evaluate the performance of quantitative CSA indices in distinguishing patients with anismus from those without, using 3D‐HRM, defecography, and EMG separately as reference standards for anismus diagnosis and applying ROC analysis. The AUC values ranged from 0.676 to 0.784 across the three anorectal investigations (Table S3, Figure S2). Quantitative CSA indices showed the highest diagnostic performance when anal EMG‐based diagnosis of anismus was used as the reference standard, with AUC values ranging from 0.722 to 0.784 (Table S3, Figure S2).

**FIGURE 4:**
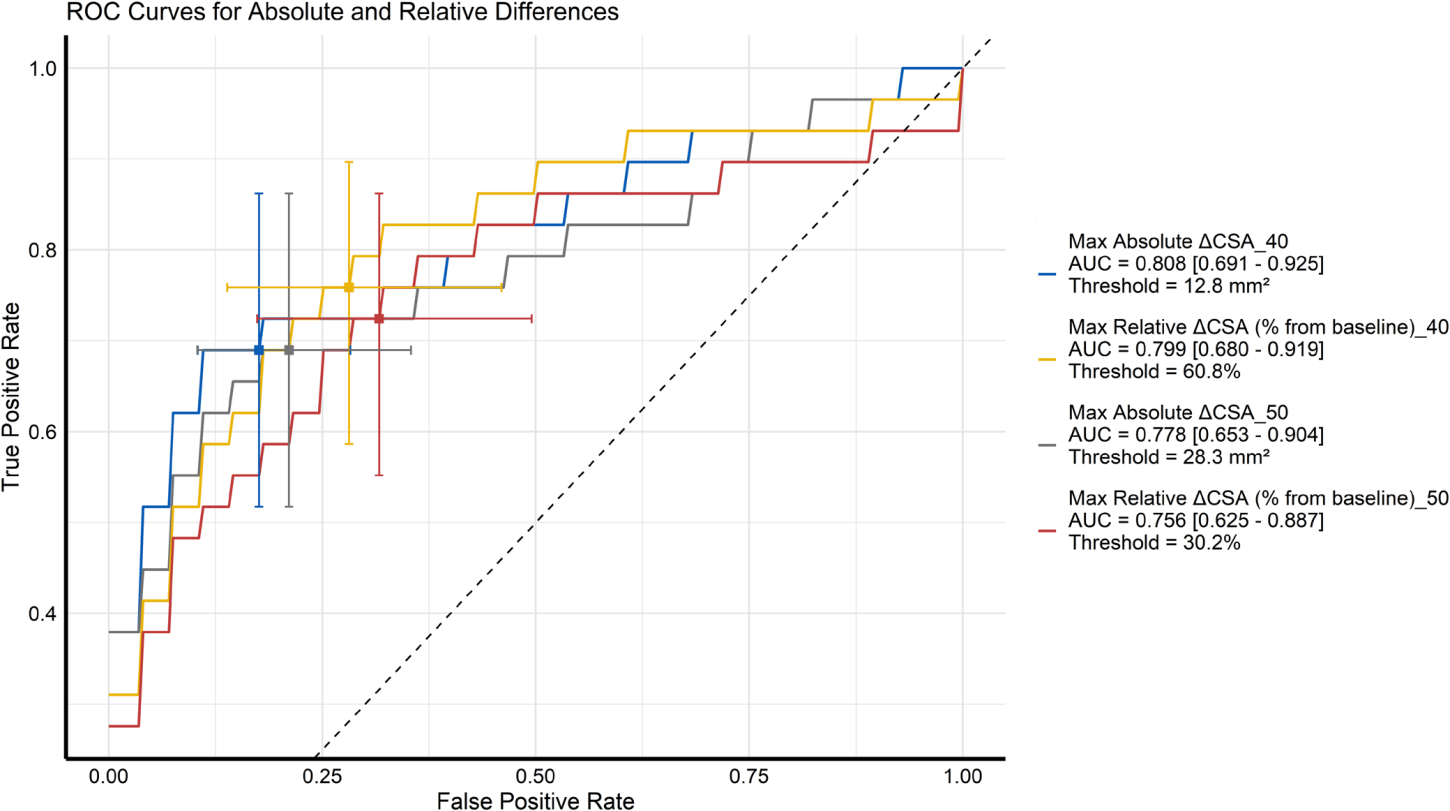
ROC curves of anismus diagnoses for CSA absolute and relative variations at 40 mL and 50 mL of distension. CSA, cross‐sectional area.

Lastly, two‐variable GLM and constrained CART models were evaluated using nested repeated cross‐validation. At 40 mL, the mean outer‐cross‐validated AUC was 0.767 (empirical 2.5th–97.5th percentiles: 0.696–0.825) for the GLM and 0.764 (0.668–0.834) for CART. Corresponding accuracy, sensitivity, and specificity were 0.743, 0.763, and 0.722 for the GLM and 0.727, 0.806, and 0.646 for CART. At 50 mL, mean AUCs were 0.754 (0.699–0.810) for the GLM and 0.658 (0.571–0.736) for CART (Table S4A).

The most frequently selected predictor pairs were absolute CSA max–min difference combined with mean absolute ΔP for the 40‐mL GLM, mean relative ΔCSA combined with mean absolute ΔP for the 40‐mL CART, absolute CSA min–baseline difference combined with mean pressure for the 50‐mL GLM, and mean relative ΔCSA combined with minimal pressure for the 50‐mL CART. These pairs were selected in 23.8%, 14.8%, 21.4%, and 9.8% of outer training folds, respectively (Table S4B), and were refitted to the complete cohort solely to generate the illustrative decision surfaces shown in Figure 5.

**FIGURE 5:**
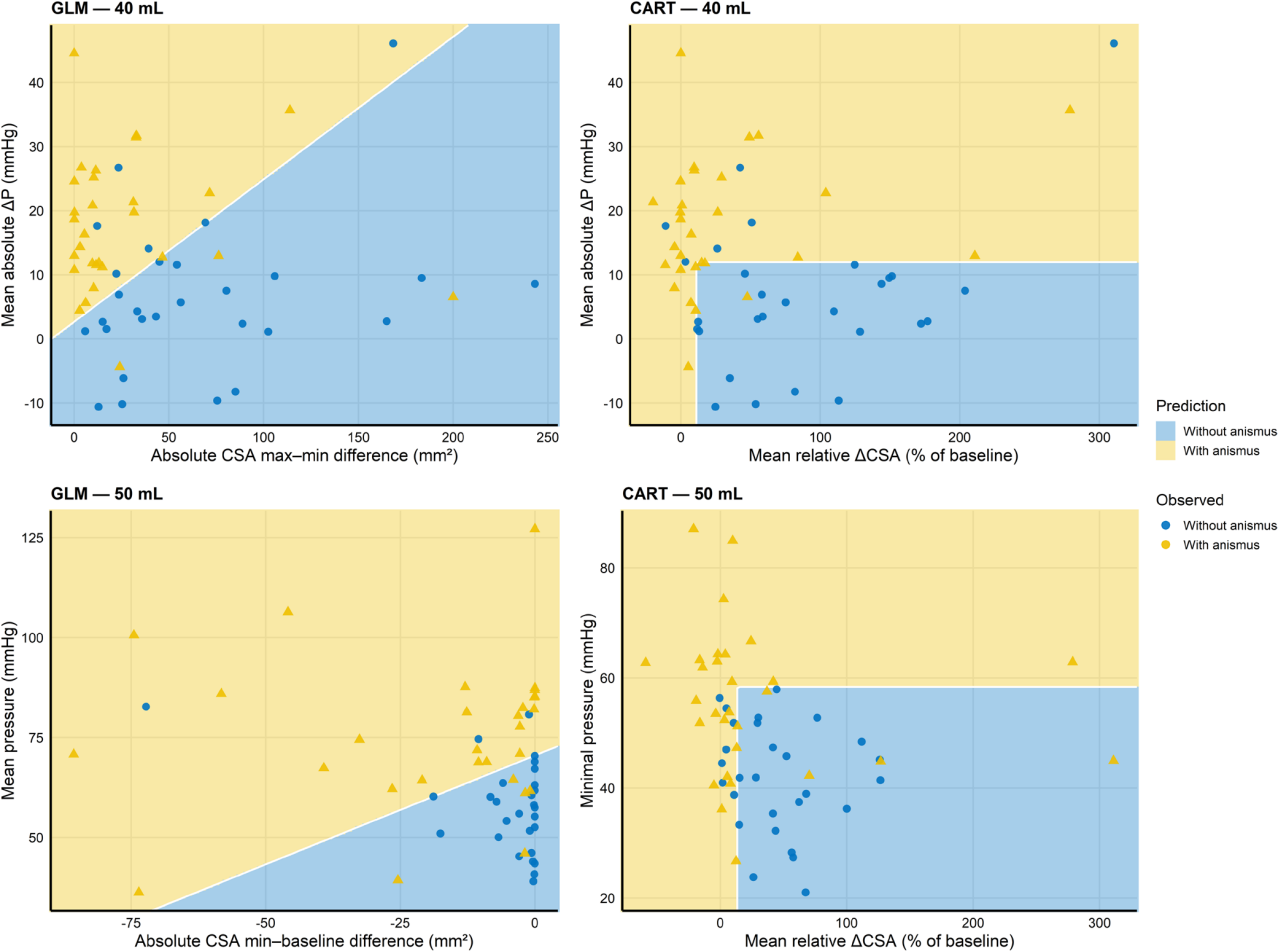
Illustrative two‐dimensional decision surfaces of generalized linear models (GLM) and constrained classification trees (CART) at 40 and 50 mL of distension. For each model and distension volume, the predictor pair most frequently selected across the 500 outer training folds of the nested repeated cross‐validation procedure was refitted to the complete patient dataset to generate the displayed decision surface. CART models were constrained to exactly two sequential axis‐aligned binary splits and to use both the CSA‐derived and pressure‐derived predictors. Background shading represents the predicted class, whereas points represent the observed diagnostic class. These full‐data fits are presented for visualization only; diagnostic performance was estimated exclusively from outer out‐of‐fold predictions obtained during nested cross‐validation. CSA, cross‐sectional area.

## 4 Discussion

The aim of this exploratory study was to evaluate the diagnostic accuracy of anal EndoFLIP to diagnose anismus in a population of constipated patients. Visual assessments yielded the same overall accuracy at both distension volumes (73.7%). However, more accurate parameters were provided by building CSA‐pressure loops during push maneuvers. An exploratory threshold under 12.8 mm^2^ for the maximal absolute ΔCSA at 40 mL achieved the highest accuracy and AUC (0.81) as well as excellent specificity (0.93) of all the quantitative CSA indices for diagnosing anismus. When predictor selection was incorporated within nested repeated cross‐validation, two‐variable models showed more conservative out‐of‐sample performance than initially estimated. At 40 mL, both GLM and CART achieved similar mean AUCs of 0.767 and 0.764, respectively, whereas the 50‐mL GLM achieved an AUC of 0.754 and the 50‐mL CART showed lower discrimination (AUC = 0.658). These internally validated estimates more appropriately reflect the uncertainty associated with screening a large number of candidate CSA–pressure combinations in a relatively small cohort.

To achieve our objectives, we used the method that we had recently developed in HVs to assess anal canal opening during push maneuvers [16]. Given the exploratory nature of the present study, which aimed to determine which parameter or combination of parameters would best discriminate patients with anismus from those without anismus, we decided to retain a large set of parameters in the final analysis. Although some parameters were not independent (e.g., those describing the opening of the anal canal during push maneuver), we wished to explore different ways of characterizing these changes. For instance, if a patient exhibited paradoxical closure of the anal canal during the push maneuver, the baseline CSA value would be greater than the minimum CSA value. Consequently, this phenomenon would not be reflected by the Max ΔCSA parameter, but it could be captured by the min‐baseline difference or the max‐min difference. Moreover, as anal EndoFLIP provides a dynamic assessment of anorectal function, we wished to highlight that parameters such as isolated maximum or mean values may be less informative in this type of analysis. In addition, we have applied the Benjamini‐Hochberg procedure to control the false discovery rate across all CSA and pressure derived comparisons.

A comparison of the characteristics of CSA‐pressure loops during push maneuver in constipated patients with or without anismus revealed that patients with anismus exhibited reduced anal canal opening (i.e., maximal and mean absolute or relative variations in CSA) and higher intra‐balloon pressures than others. These results were expected and were in line with those from prior studies using 3D‐HRM in patients with anismus. Indeed, anismus is described in the literature as a default of anal relaxation or a paradoxical increase in anal pressure during push maneuver [16]. Based on the potential markers of anal canal opening that we identified in HVs [32], an exploratory threshold of 12.8 mm^2^ for the maximal absolute ΔCSA provided the most accurate AUC (0.81) for discriminating patients with anismus from those without. In the prior study in HVs, the parameter assessing anal canal opening with the lowest variability among HVs was the mean relative ΔCSA but gave a slightly lower AUC (0.79) in the present study. Among the two‐variable models, the 40‐mL GLM and CART showed the highest internally validated discrimination. For the 40‐mL GLM, the most frequently selected predictor combination comprised the absolute CSA max–min difference and mean absolute pressure variation, which is physiologically plausible because these parameters characterize complementary components of the push maneuver, namely anal canal opening and pressure generation. However, this exact combination was selected in 23.8% of outer training folds. Predictor selection was concentrated on a subset of CSA–pressure combinations, although no single exact pair overwhelmingly dominated across resamples. The most frequently selected pairs were retained in 9.8%–23.8% of outer training folds, which likely reflects, at least in part, the substantial redundancy and correlation among several derived CSA and pressure parameters. However, it should be noted that the intra‐balloon pressure sensor of the anal EndoFLIP catheter is located at its distal‐end. Consequently, the occurrence of high‐pressure variations during push maneuvers could indicate an exaggerated increase in rectal pressure that could be attributable to abnormal abdominal thrusts in the event of an anal opening default, or to an increase in anal pressure due to paradoxical closure of anal canal. This complicates the interpretation of our given pressure‐based classifier. For this reason, anal EndoFLIP, in its current configuration, does not provide a complete assessment of rectal propulsive forces. In contrast, parameters reflecting anal canal opening, such as the maximal–minimal CSA difference, were expected to be altered in patients with anismus. Therefore, although EndoFLIP may provide complementary diagnostic information, its findings should not be interpreted in isolation but rather in conjunction with a comprehensive assessment of defecatory function.

To our knowledge, only a few studies have compared the performances of different anorectal tests for diagnosing dyssynergic defecation, and they were not specifically performed with patients with suspected anismus in most cases [33]. In the present study, 3D‐HRM provided the highest rate of diagnosis of anismus (65.0%), followed by anal EMG (49.2%) and defecography (22.2%). A final diagnosis (at least two positive examinations) was reached in 50.8% of patients. Differences in the rates of anismus diagnosis highlight the discrepancy between these tests. The high concordance between HR‐ARM and anal EMG observed in the present study should also be noted, as impaired rectal evacuation was not demonstrated in most patients. This may affect the interpretation of the diagnostic accuracy of EndoFLIP, which, in the present study, was based on impaired anal relaxation rather than impaired rectal evacuation. As no parameter is currently favored for diagnosing anismus on 3D‐HRM in the guidelines, we chose to use types of anismus rather than rectoanal gradient in the present study [33]. Indeed, types of dyssynergic defecation include interpretations of anal relaxation or paradoxical contraction apart from the increase in rectal pressure, as rectoanal gradient can be abnormal when isolated ineffective propulsion has been diagnosed [5]. The parameters used to diagnose anismus by anal EMG and defecography in the present study have been previously described by others [24, 26, 33]. Lastly, as interpretations of rectal pressure variations on anal EndoFLIP results must be performed carefully, we did not choose to assess these variations to diagnose ineffective propulsion. The authors acknowledge that focusing solely on anismus, without assessing rectal propulsive forces, as is currently possible with anal EndoFLIP, may add another test to an already broad landscape of complementary investigations, particularly as a unique and definitive gold standard for the assessment of anorectal function has yet to be established.

Although these results are promising, they require confirmation in further studies to determine the potential place of anal EndoFLIP within an algorithm of anorectal physiological tests for diagnosing dyssynergic defecation. At present, it seems problematic to use only one anorectal physiological test in this case as each test explores different dimensions of the defecation process [34]. For its part, EndoFLIP evaluates resistance to the distension of the anal sphincter, which cannot be evaluated by common anorectal physiological tests such as 3D‐HRM [35]. Indeed, it has been suggested that resistance to distension could probably better reflect an obstacle in the anal canal [35]. The quantitative CSA parameters observed in the present study (i.e., maximal or mean absolute and relative ΔCSAs) described anal canal opening and, by extension, increased resistance to stool passage during defecation as expected in anismus. However, we cannot draw clear conclusions from this exploratory study, for example, to determine whether anal EndoFLIP can bring additional meaningful metrics to conventional anorectal explorations. Moreover, another area of research for anal EndoFLIP would be to test the clinical utility of this anorectal physiological test, especially its predictive value for determining the efficacy of biofeedback/pelvic floor physical therapy. In the present study, a prospective follow‐up after the performance of anorectal physiological tests was not initially planned; therefore, we unfortunately cannot answer this specific question. Indeed, a recent study reported that BET, as a single test, or 3D‐HRM results during push maneuver, might not be the best parameters for predicting treatment outcomes, unlike squeeze profiles [36]. This topic will be the subject of future research.

Lastly, the present study had some limitations. Firstly, the sample size was small, the study included only patients with constipation and suspected defecatory dysfunction, and we did not include a healthy control group. This would have strengthened the study, due to the low specificity of ARM to diagnose anismus, and due to the disagreement between anorectal physiological tests. Consequently, our findings cannot determine whether the proposed exploratory thresholds can distinguish patients with anismus from individuals with normal anorectal physiology or other pelvic‐floor disorders. As previously stated, our composite reference standard was not consistent with current guidelines, as we did not perform BET to evaluate rectal evacuation as part of our composite reference standard for several reasons, despite the fact that this test is currently widely used in the medical community due to its ease of use and low cost [33]. Firstly, the BET only assesses rectal evacuation, not the evolution of anorectal parameters during defecation, representing a limit for diagnosing anismus [8, 25]. Instead, we chose to use defecography, which allows assessment of both rectal evacuation and the anal opening, as previously published for diagnosing anismus [24]. Moreover, a normal BET result does not exclude the presence of dyssynergic defecation on defecography [37]. BET may be normal in patients with dyssynergic defecation who are able to compensate by excessive straining [19]. It has also been demonstrated that using a fixed‐volume BET to achieve constant desire to defecate volume as currently recommended could overdiagnose dyssynergic defecation [8, 38]. Lastly, there is still conflicting results regarding the predictive response to biofeedback on basis of BET results [39, 40]. In addition, colonic transit study was used as a screening tool to exclude patients with slow transit constipation alone in the present study. We acknowledge this limitation, as it has been evidenced that patients with evidence of dyssynergic defecation were no more likely to have rectosigmoid retention of markers than those without [41]. On the contrary, it also has been shown that colonic transit time can be delayed in patients with either slow transit constipation or evacuation disorders, or both conditions [42]. Despite the use of nested repeated cross‐validation, the large number of candidate predictor combinations relative to the sample size remains a limitation. Although predictor selection was clearly non‐uniform across resamples, no single exact CSA–pressure pair overwhelmingly dominated, likely reflecting the substantial correlation and redundancy among several derived parameters. Therefore, the two‐variable models should be considered exploratory, and both their diagnostic performance and predictor selection require confirmation in an independent external cohort. Lastly, the EndoFLIP software does not currently allow the selection of ROIs and the export of linked data. This is why we used software specifically designed for this type of analysis. In addition, as the EndoFLIP probe only contains a single intra‐balloon pressure sensor, we chose to focus mainly on CSA variations during push maneuvers. Indeed, rectal propulsive forces cannot be clearly computed from it unlike with HR‐ARM and defecography.

## 5 Conclusion

By building CSA–pressure loops during push maneuvers, we found that a threshold under 12.8 mm^2^ for the maximal absolute ΔCSA at 40 mL achieved the highest accuracy and AUC (0.81), as well as excellent specificity (0.93) for diagnosing anismus among quantitative CSA indices. Two‐variable CSA–pressure models provided mean nested cross‐validated AUCs ranging from 0.658 to 0.767, with the highest discrimination observed at 40 mL. These exploratory models require independent external validation and further confirmatory studies are needed to establish clinically applicable thresholds.

## Author Contributions

C.D. and A.‐M.L. designed the study; C.D., A.‐M.L., G.G., C.M., and C.S.‐C. included patients and collected data; C.D., A.‐M.L., T.D., and E.H. analyzed and interpreted data; C.D., A.‐M.L., and T.D. drafted the manuscript; C.D., T.D., A.‐M.L., G.G., C.M., A.Z., C.S.‐C., and E.H. revised the manuscript and approved the final version of the manuscript to be published. All authors have approved the final version of the article, including the authorship list.

## Conflicts of Interest

A.Z. holds patent rights to the EndovizX software used in the present study. C.M. has served as a consultant for Kyowan Kirin, Norgine, Biocodex, Mayoly Spindler, Tillots, Ipsen, Nestlé, HealthScience, Viatris, Reckitt Benckiser Health Limited, and De Opella. The other authors have no conflicts of interest to declare.

## Supporting information


**Figure S1:** Distribution of absolute and relative variations in CSA (baseline minus minimum CSA) by distension volume and anismus diagnosis status. CSA: cross‐sectional‐area.
**Figure S2:** ROC curves of anismus diagnoses by ARM (A), defecography (B) and anal EMG (C) for CSA absolute and relative variations at 40 mL and 50 mL of distension. ARM, anorectal manometry; CSA, cross‐sectional area; EMG, electromyography.

**Table S1:** Discriminative performance for the diagnosis of anismus of 48 CSA‐pressure loop characteristics at 40 mL and 50 mL of distension using a ROC analysis.
**Table S2:** Correlation analyses between EndoFLIP parameters and GIQLI and Kess scores at 40 and 50 mL of distension.
**Table S3:** ROC analysis of the performance of quantitative CSA indices for distinguishing patients with anismus from those without, using ARM, defecography, and EMG as separate reference standards.
**Table S4:** (A) Out‐of‐sample performance of two‐variable EndoFLIP classifiers estimated using nested repeated cross‐validation. Values are means across 100 repetitions; values in brackets represent the empirical 2.5th–97.5th percentiles across repetitions and should not be interpreted as formal confidence intervals. (B) Most frequently selected CSA–pressure predictor pairs across the nested repeated cross‐validation procedure. Selection frequency represents the proportion of the 500 outer training folds (5 folds × 100 repetitions) in which a given CSA–pressure pair achieved the highest inner out‐of‐fold AUC for the corresponding model and distension volume. The displayed pair was the most frequently selected pair for each GLM/CART and volume combination and was refitted to the complete patient dataset only for the illustrative decision surfaces shown in Figure 5. Selection frequency should be interpreted as a measure of predictor‐pair stability rather than diagnostic performance.

## Data Availability

Raw data (deidentified individual participant data), the R code, and the resulting variable outputs will be made available upon reasonable request at www.figshare.com. Requests for access should be sent to charlotte-desprez@orange.fr.
