# Supplementary material for: Diagnostic Value of the Anal Endoluminal Functional Luminal Imaging Probe for the Diagnosis of Anismus: An Exploratory Study
Source: Neurogastroenterol Motil. 2026 Oct 2;38(10):e70452. doi: 10.1111/nmo.70452 (PMC13631694; doi:10.1111/nmo.70452)
Supplement: Supplementary file 1 [file NMO-38-e70452-s002.docx]

**SUPPLEMENTARY TABLES**

**Table S1: Discriminative performance for the diagnosis of anismus of 48 CSA-pressure loop characteristics at 40 mL and 50 mL of distension using a ROC analysis**

| **Predictor** | **AUC** | **Threshold** | **Sensitivity** | **Specificity** | **Accuracy** |
| --- | --- | --- | --- | --- | --- |
| Mean Pressure_50 | 0.815 | 64.0 mmHg | 0.793 | 0.786 | 0.789 |
| Mean Absolute ΔP_40 | 0.810 | 10.5 mmHg | 0.828 | 0.750 | 0.789 |
| Max Absolute ΔCSA_40 | 0.808 | 12.8 mm² | 0.690 | 0.929 | 0.807 |
| Max Relative ΔCSA (% from baseline)_40 | 0.799 | 60.8% | 0.759 | 0.786 | 0.772 |
| Absolute CSA max-min difference_40 | 0.798 | 11.9 mm² | 0.586 | 0.964 | 0.772 |
| Mean Pressure_40 | 0.797 | 58.2 mmHg | 0.724 | 0.821 | 0.772 |
| Relative CSA min-max difference (% from min)_40 | 0.796 | 72.9% | 0.793 | 0.750 | 0.772 |
| Min Pressure_50 | 0.794 | 53.1 mmHg | 0.586 | 0.893 | 0.737 |
| Mean Relative ΔCSA (% from baseline)_40 | 0.793 | 11.2% | 0.621 | 0.929 | 0.772 |
| Mean Absolute ΔCSA_40 | 0.791 | 8.2 mm² | 0.724 | 0.893 | 0.807 |
| Absolute P baseline-min difference_40 | 0.784 | -8.2 mmHg | 0.931 | 0.607 | 0.772 |
| Relative P min-baseline difference (% from min)_40 | 0.781 | -19.9% | 0.897 | 0.607 | 0.754 |
| Max Absolute ΔCSA_50 | 0.778 | 28.3 mm² | 0.690 | 0.857 | 0.772 |
| Mean Absolute ΔCSA_50 | 0.776 | 10.1 mm² | 0.655 | 0.857 | 0.754 |
| Min Pressure_40 | 0.775 | 41.1 mmHg | 0.690 | 0.786 | 0.737 |
| Mean Relative ΔP (% from baseline)_40 | 0.775 | 26.6% | 0.724 | 0.750 | 0.737 |
| Mean Absolute ΔP_50 | 0.774 | 8.6 mmHg | 0.828 | 0.679 | 0.754 |
| Max CSA_40 | 0.767 | 37.2 mm² | 0.517 | 0.964 | 0.737 |
| Max Pressure_50 | 0.762 | 79.6 mmHg | 0.621 | 0.857 | 0.737 |
| Max Relative ΔCSA (% from baseline)_50 | 0.756 | 30.2% | 0.724 | 0.750 | 0.737 |
| Mean CSA_40 | 0.754 | 37.6 mm² | 0.586 | 0.893 | 0.737 |
| Mean Relative ΔCSA (% from baseline)_50 | 0.754 | 14.3% | 0.759 | 0.750 | 0.754 |
| Max Pressure_40 | 0.750 | 66.2 mmHg | 0.724 | 0.750 | 0.737 |
| Relative P min-baseline difference (% from min)_50 | 0.726 | -6.5% | 0.828 | 0.643 | 0.737 |
| Relative CSA min-baseline difference (% from min)_50 | 0.725 | -1.0% | 0.793 | 0.607 | 0.702 |
| Absolute P min-baseline difference_50 | 0.721 | -3.4 mmHg | 0.828 | 0.643 | 0.737 |
| Mean Relative ΔP (% from baseline)_50 | 0.717 | 11.6% | 0.897 | 0.571 | 0.737 |
| Max Absolute ΔP_50 | 0.711 | 15.9 mmHg | 0.759 | 0.643 | 0.702 |
| Absolute CSA min-baseline difference_50 | 0.706 | -8.6 mm² | 0.517 | 0.857 | 0.684 |
| Max Absolute ΔP_40 | 0.701 | 22.0 mmHg | 0.655 | 0.679 | 0.667 |
| Absolute CSA max-min difference_50 | 0.697 | 44.5 mm² | 0.621 | 0.786 | 0.702 |
| Max CSA_50 | 0.693 | 137.2 mm² | 0.586 | 0.786 | 0.684 |
| Mean CSA_50 | 0.690 | 133.1 mm² | 0.586 | 0.786 | 0.684 |
| Baseline Pressure_50 | 0.677 | 59.8 mmHg | 0.448 | 0.893 | 0.667 |
| Min CSA_40 | 0.669 | 21.6 mm² | 0.448 | 0.893 | 0.667 |
| Max Relative ΔP (% from baseline)_40 | 0.662 | 34.6% | 0.793 | 0.500 | 0.649 |
| Max Relative ΔP (% from baseline)_50 | 0.651 | 29.9% | 0.690 | 0.607 | 0.649 |
| Min CSA_50 | 0.650 | 88.2 mm² | 0.586 | 0.750 | 0.667 |
| Baseline CSA_40 | 0.649 | 26.4 mm² | 0.517 | 0.786 | 0.649 |
| Baseline Pressure_40 | 0.629 | 41.0 mmHg | 0.793 | 0.500 | 0.649 |
| Relative CSA min-max difference (% from min)_50 | 0.610 | 56.7% | 0.690 | 0.643 | 0.667 |
| Relative P min-max difference (% from min)_40 | 0.605 | 111.3% | 0.931 | 0.357 | 0.649 |
| Baseline CSA_50 | 0.594 | 73.5 mm² | 0.345 | 0.893 | 0.614 |
| Absolute P max-min difference_50 | 0.579 | 30.6 mmHg | 0.448 | 0.786 | 0.614 |
| Relative P min-max difference (% from min)_50 | 0.568 | 83.8% | 0.862 | 0.357 | 0.614 |
| Absolute CSA baseline-min difference_40 | 0.504 | -0.4 mm² | 0.621 | 0.536 | 0.579 |
| Relative CSA min-baseline difference (% from min)_40 | 0.491 | -2.1% | 0.690 | 0.464 | 0.579 |
| Absolute P max-min difference_40 | 0.469 | 25.6 mmHg | 0.517 | 0.571 | 0.544 |

AUC: area under the curve; CSA: cross-sectional-area (mm^2^); P: pressure (mmHg); 40: 40 mL of distension, 50: 50 mL of distension

**Table S2: Correlation analyses between EndoFLIP^TM^ parameters and GIQLI and Kess scores at 40 and 50 mL of distension**

| **EndoFLIP parameter** | | **Score** | **n** | **Spearman r** | | | **Raw p-value** | **BH-adjusted p-value** | |  |
| --- | --- | --- | --- | --- | --- | --- | --- | --- | --- | --- |
| Maximum absolute ΔCSA_40 (mm²) | | GIQLI | 51 | -0.146 | | | 0.307 | 0.639 | |  |
| Maximum relative ΔCSA_40 (% of baseline) | | GIQLI | 51 | -0.151 | | | 0.291 | 0.639 | |  |
| Mean absolute ΔCSA_40 (mm²) | | GIQLI | 51 | -0.058 | | | 0.685 | 0.822 | |  |
| Mean relative ΔCSA_40 (% of baseline) | | GIQLI | 51 | -0.086 | | | 0.550 | 0.777 | |  |
| Absolute CSA min-baseline difference_40 (mm²) | | GIQLI | 51 | 0.027 | | | 0.852 | 0.852 | |  |
| Relative CSA min-baseline difference_40 (%) | | GIQLI | 51 | 0.029 | | | 0.841 | 0.852 | |  |
| Absolute CSA max-min difference_40 (mm²) | | GIQLI | 51 | -0.147 | | | 0.304 | 0.639 | |  |
| Relative CSA max-min difference_40 (%) | | GIQLI | 51 | -0.148 | | | 0.300 | 0.639 | |  |
| Maximum absolute ΔP_40 (mmHg) | | GIQLI | 51 | -0.188 | | | 0.188 | 0.639 | |  |
| Mean absolute ΔP_40 (mmHg) | | GIQLI | 51 | -0.287 | | | 0.041 | 0.497 | |  |
| Mean relative ΔP_40 (% of baseline) | | GIQLI | 51 | -0.313 | | | 0.025 | 0.497 | |  |
| Absolute pressure min-baseline difference_40 (mmHg) | | GIQLI | 51 | -0.113 | | | 0.432 | 0.691 | |  |
| Relative pressure min-baseline difference_40 (%) | | GIQLI | 51 | -0.088 | | | 0.541 | 0.777 | |  |
| Maximum absolute ΔCSA_40 (mm²) | | KESS | 51 | -0.148 | | | 0.299 | 0.478 | |  |
| Maximum relative ΔCSA_40 (% of baseline) | | KESS | 51 | -0.197 | | | 0.166 | 0.416 | |  |
| Mean absolute ΔCSA_40 (mm²) | | KESS | 51 | -0.141 | | | 0.325 | 0.487 | |  |
| Mean relative ΔCSA_40 (% of baseline) | | KESS | 51 | -0.183 | | | 0.198 | 0.416 | |  |
| Absolute CSA min-baseline difference_40 (mm²) | | KESS | 51 | -0.176 | | | 0.217 | 0.416 | |  |
| Relative CSA min-baseline difference_40 (%) | | KESS | 51 | -0.202 | | | 0.155 | 0.416 | |  |
| Absolute CSA max-min difference_40 (mm²) | | KESS | 51 | -0.082 | | | 0.567 | 0.716 | |  |
| Relative CSA max-min difference_40 (%) | | KESS | 51 | -0.113 | | | 0.430 | 0.607 | |  |
| Maximum absolute ΔP_40 (mmHg) | | KESS | 51 | 0.321 | | | 0.022 | 0.249 | |  |
| Mean absolute ΔP_40 (mmHg) | | KESS | 51 | 0.296 | | | 0.035 | 0.249 | |  |
| Mean relative ΔP_40 (% of baseline) | | KESS | 51 | 0.267 | | | 0.058 | 0.249 | |  |
| Absolute pressure min-baseline difference_40 (mmHg) | | KESS | 51 | 0.205 | | | 0.148 | 0.416 | |  |
| Relative pressure min-baseline difference_40 (%) | | KESS | 51 | 0.172 | | | 0.229 | 0.416 | |  |
|  | Maximum absolute ΔCSA_50 (mm²) | GIQLI | 51 | | -0.055 | 0.704 | | | 0.896 | |
|  | Maximum relative ΔCSA_50 (% of baseline) | GIQLI | 51 | | -0.019 | 0.896 | | | 0.896 | |
|  | Mean absolute ΔCSA_50 (mm²) | GIQLI | 51 | | 0.026 | 0.857 | | | 0.896 | |
|  | Mean relative ΔCSA_50 (% of baseline) | GIQLI | 51 | | 0.027 | 0.851 | | | 0.896 | |
|  | Absolute CSA min-baseline difference_50 (mm²) | GIQLI | 51 | | 0.220 | 0.122 | | | 0.720 | |
|  | Relative CSA min-baseline difference_50 (%) | GIQLI | 51 | | 0.236 | 0.095 | | | 0.720 | |
|  | Absolute CSA max-min difference_50 (mm²) | GIQLI | 51 | | -0.124 | 0.386 | | | 0.772 | |
|  | Relative CSA max-min difference_50 (%) | GIQLI | 51 | | -0.151 | 0.292 | | | 0.754 | |
|  | Maximum absolute ΔP_50 (mmHg) | GIQLI | 51 | | -0.198 | 0.163 | | | 0.720 | |
|  | Mean absolute ΔP_50 (mmHg) | GIQLI | 51 | | -0.167 | 0.240 | | | 0.720 | |
|  | Mean relative ΔP_50 (% of baseline) | GIQLI | 51 | | -0.176 | 0.217 | | | 0.720 | |
|  | Absolute pressure min-baseline difference_50 (mmHg) | GIQLI | 51 | | 0.027 | 0.851 | | | 0.896 | |
|  | Relative pressure min-baseline difference_50 (%) | GIQLI | 51 | | 0.021 | 0.886 | | | 0.896 | |
|  | Maximum absolute ΔCSA_50 (mm²) | KESS | 51 | | -0.064 | 0.656 | | | 0.716 | |
|  | Maximum relative ΔCSA_50 (% of baseline) | KESS | 51 | | -0.087 | 0.542 | | | 0.716 | |
|  | Mean absolute ΔCSA_50 (mm²) | KESS | 51 | | -0.083 | 0.562 | | | 0.716 | |
|  | Mean relative ΔCSA_50 (% of baseline) | KESS | 51 | | -0.080 | 0.577 | | | 0.716 | |
|  | Absolute CSA min-baseline difference_50 (mm²) | KESS | 51 | | -0.168 | 0.238 | | | 0.569 | |
|  | Relative CSA min-baseline difference_50 (%) | KESS | 51 | | -0.191 | 0.180 | | | 0.526 | |
|  | Absolute CSA max-min difference_50 (mm²) | KESS | 51 | | -0.052 | 0.717 | | | 0.718 | |
|  | Relative CSA max-min difference_50 (%) | KESS | 51 | | -0.052 | 0.718 | | | 0.718 | |
|  | Maximum absolute ΔP_50 (mmHg) | KESS | 51 | | 0.277 | 0.049 | | | 0.393 | |
|  | Mean absolute ΔP_50 (mmHg) | KESS | 51 | | 0.224 | 0.115 | | | 0.526 | |
|  | Mean relative ΔP_50 (% of baseline) | KESS | 51 | | 0.160 | 0.261 | | | 0.569 | |
|  | Absolute pressure min-baseline difference_50 (mmHg) | KESS | 51 | | 0.112 | 0.432 | | | 0.716 | |
|  | Relative pressure min-baseline difference_50 (%) | KESS | 51 | | 0.128 | 0.371 | | | 0.716 | |

**Table S3: ROC analysis of the performance of quantitative CSA indices for distinguishing patients with anismus from those without, using ARM, defecography, and EMG as separate reference standards**

| Predictor | Anismus diagnosis | AUC | Sensitivity | Specificity |
| --- | --- | --- | --- | --- |
| Max Absolute ΔCSA_40 | ARM | 0.676 | 0.658 | 0.667 |
| Max Relative ΔCSA (% from baseline)_40 | ARM | 0.694 | 0.763 | 0.619 |
| Max Absolute ΔCSA_50 | ARM | 0.727 | 0.631 | 0.810 |
| Max Relative ΔCSA (% from baseline)_50 | ARM | 0.711 | 0.684 | 0.810 |
| Max Absolute ΔCSA_40 | Defecography | 0.730 | 0.667 | 0.756 |
| Max Relative ΔCSA (% from baseline)_40 | Defecography | 0.754 | 0.917 | 0.560 |
| Max Absolute ΔCSA_50 | Defecography | 0.691 | 0.750 | 0.685 |
| Max Relative ΔCSA (% from baseline)_50 | Defecography | 0.679 | 0.833 | 0.634 |
| Max Absolute ΔCSA_40 | EMG | 0.786 | 0.733 | 0.833 |
| Max Relative ΔCSA (% from baseline)_40 | EMG | 0.769 | 0.800 | 0.700 |
| Max Absolute ΔCSA_50 | EMG | 0.747 | 0.700 | 0.800 |
| Max Relative ΔCSA (% from baseline)_50 | EMG | 0.722 | 0.867 | 0.533 |

ARM: anorectal manometry; EMG: electromyography

**Tables S4:**

**Table S4A.** **Out-of-sample performance of two-variable EndoFLIP classifiers estimated using nested repeated cross-validation**. Values are means across 100 repetitions; values in brackets represent the empirical 2.5th–97.5th percentiles across repetitions and should not be interpreted as formal confidence intervals.

| **Volume (mL)** | **Model** | **AUC** | **Accuracy** | **Sensitivity** | **Specificity** |
| --- | --- | --- | --- | --- | --- |
| 40 | GLM | 0.767 [0.696–0.825] | 0.743 [0.667–0.825] | 0.763 [0.690–0.862] | 0.722 [0.624–0.821] |
| 40 | CART | 0.764 [0.668–0.834] | 0.727 [0.632–0.807] | 0.806 [0.655–0.931] | 0.646 [0.481–0.769] |
| 50 | GLM | 0.754 [0.699–0.810] | 0.720 [0.649–0.789] | 0.702 [0.586–0.793] | 0.739 [0.607–0.857] |
| 50 | CART | 0.658 [0.571–0.736] | 0.643 [0.552–0.711] | 0.695 [0.552–0.828] | 0.589 [0.429–0.733] |

**Table S4B. Most frequently selected CSA–pressure predictor pairs across the nested repeated cross-validation procedure.** Selection frequency represents the proportion of the 500 outer training folds (5 folds × 100 repetitions) in which a given CSA–pressure pair achieved the highest inner out-of-fold AUC for the corresponding model and distension volume. The displayed pair was the most frequently selected pair for each GLM/CART and volume combination and was refitted to the complete patient dataset only for the illustrative decision surfaces shown in Figure 5. Selection frequency should be interpreted as a measure of predictor-pair stability rather than diagnostic performance.

| **Volume (mL)** | **Model** | **CSA variable** | **Pressure variable** | **Selection frequency** |
| --- | --- | --- | --- | --- |
| 40 | GLM | Absolute CSA max–min difference (mm²) | Mean absolute ΔP (mmHg) | 23.8% |
| 40 | CART | Mean relative ΔCSA (% of baseline) | Mean absolute ΔP (mmHg) | 14.8% |
| 50 | GLM | Absolute CSA min–baseline difference (mm²) | Mean pressure (mmHg) | 21.4% |
| 50 | CART | Mean relative ΔCSA (% of baseline) | Minimal pressure (mmHg) | 9.8% |
