# Supplementary material for: Diagnostic Value of the Anal Endoluminal Functional Luminal Imaging Probe for the Diagnosis of Anismus: An Exploratory Study
Source: Neurogastroenterol Motil. 2026 Oct 2;38(10):e70452. doi: 10.1111/nmo.70452 (PMC13631694; doi:10.1111/nmo.70452)
Supplement: Supplementary file 2 [file NMO-38-e70452-s001.zip › nmo70452-sup-0003-FigureS2@Figure S2.pdf]

Figure S2

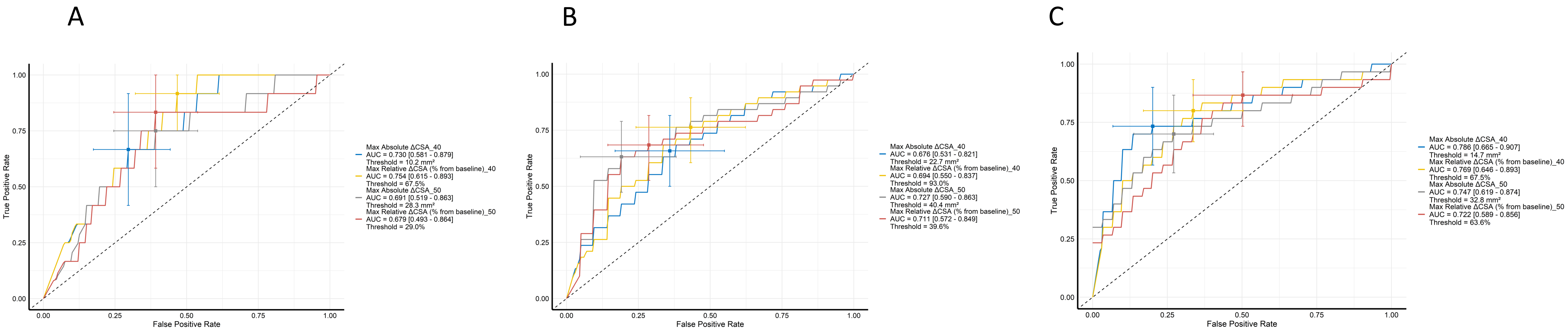
A
True Positive Rate
False Positive Rate
Max Absolute ΔCSA_40
AUC = 0.730 [0.581 - 0.879]
Threshold = 10.2 mm²
Max Relative ΔCSA (% from baseline)_40
AUC = 0.754 [0.615 - 0.893]
Threshold = 67.5%
Max Absolute ΔCSA_50
AUC = 0.691 [0.519 - 0.863]
Threshold = 28.3 mm²
Max Relative ΔCSA (% from baseline)_50
AUC = 0.679 [0.493 - 0.864]
Threshold = 29.0%
B
True Positive Rate
False Positive Rate
Max Absolute ΔCSA_40
AUC = 0.676 [0.531 - 0.821]
Threshold = 22.7 mm²
Max Relative ΔCSA (% from baseline)_40
AUC = 0.694 [0.550 - 0.837]
Threshold = 93.0%
Max Absolute ΔCSA_50
AUC = 0.727 [0.590 - 0.863]
Threshold = 40.4 mm²
Max Relative ΔCSA (% from baseline)_50
AUC = 0.711 [0.572 - 0.849]
Threshold = 39.6%
C
True Positive Rate
False Positive Rate
Max Absolute ΔCSA_40
AUC = 0.786 [0.665 - 0.907]
Threshold = 14.7 mm²
Max Relative ΔCSA (% from baseline)_40
AUC = 0.769 [0.646 - 0.893]
Threshold = 67.5%
Max Absolute ΔCSA_50
AUC = 0.747 [0.619 - 0.874]
Threshold = 32.8 mm²
Max Relative ΔCSA (% from baseline)_50
AUC = 0.722 [0.589 - 0.856]
Threshold = 63.6%
