# Supplementary material for: Diagnostic Value of the Anal Endoluminal Functional Luminal Imaging Probe for the Diagnosis of Anismus: An Exploratory Study
Source: Neurogastroenterol Motil. 2026 Oct 2;38(10):e70452. doi: 10.1111/nmo.70452 (PMC13631694; doi:10.1111/nmo.70452)
Supplement: Supplementary file 2 [file NMO-38-e70452-s001.zip › nmo70452-sup-0002-FigureS1-S2@SUPPLEMENTARY FIGURES LEGEND.docx]

**SUPPLEMENTARY FIGURES**

**Figure S1: Distribution of absolute and relative variations in CSA (baseline minus minimum CSA) by distension volume and anismus diagnosis status.** CSA: cross-sectional-area.

**Figure S2: ROC curves of anismus diagnoses by ARM (A), defecography (B) and anal EMG (C) for CSA absolute and relative variations at 40 mL and 50 mL of distension.** ARM: anorectal manometry, CSA: cross-sectional area, EMG: electromyography.
